# Results of a phase I, open-label, randomized, crossover study evaluating the effects of linifanib on QTc intervals in patients with solid tumors

**DOI:** 10.1007/s00280-013-2351-2

**Published:** 2013-11-16

**Authors:** Yi-Lin Chiu, Patricia LoRusso, Balakrishna Hosmane, Justin L. Ricker, Walid Awni, Dawn M. Carlson

**Affiliations:** 1 AbbVie, Inc., North Chicago, IL USA; 2 Wayne State University, Detroit, MI USA

**Keywords:** Tyrosine kinase inhibitors, QT, ECG, VEGF

## Abstract

**Purpose:**

Linifanib is a selective inhibitor of the vascular endothelial growth factor and platelet-derived growth factor family of tyrosine kinase inhibitors. The purpose of this high-precision QT study was to evaluate the effects of linifanib on cardiac repolarization in patients with advanced metastatic tumors.

**Methods:**

Enrolled patients (*n* = 24) had measurable disease refractory to standard therapies, ECOG performance status of 0–1, and adequate organ function. Patients were randomized in a 2-sequence, 2-period crossover design. Serial ECG measurements and pharmacokinetic samples were collected for each crossover period. An intersection–union test was performed for time-matched baseline-adjusted QTcF intervals. An exposure–response analysis was explored to correlate the plasma concentration and QTcF.

**Results:**

The maximum 95 % upper confidence bound for the baseline-adjusted QTcF was 4.3 ms at hour 3 at the maximum tolerated linifanib dose of 0.25 mg/kg. Linifanib did not meet the regulatory threshold (10 ms) for QT prolongation. Exposure–response modeling showed that the QTcF change was not significant at the maximum plasma concentration.

**Conclusions:**

Linifanib does not significantly affect cardiac repolarization in patients with advanced solid tumors.

## Introduction

Cardiac safety has become a vital issue for cancer patients as life expectancies are increased with emerging therapies. The ability to balance both efficacy and safety is critical for patient survival and quality of life. Cancer therapy is becoming increasingly specialized, having evolved from using cytotoxic drugs on dividing cells to targeting specific molecular events involved in oncogenic proliferation. Among these targeted therapeutics is a drug class known as tyrosine kinase inhibitors (TKIs). Tyrosine kinases are enzymes that catalyze phosphorylation of target proteins, signaling cellular processes such as growth and proliferation. Unregulated tyrosine kinase activation, therefore, can cause uncontrolled cellular proliferation, leading to cancer. Although complete inhibition of tyrosine kinases may disrupt vital cellular signaling, targeted TKIs may prevent cancerous proliferation while sparing essential kinase activity [1, 2].

Linifanib is a novel receptor TKI with specificity for the vascular endothelial growth factor (VEGF) and platelet-derived growth factor (PDGF) receptors. It does not possess significant activity against cytosolic tyrosine kinases or serine/threonine kinases [3]. As tumor progression can rely on both VEGF and PDGF signaling, a selective inhibitor could result in high antitumor activity without interrupting other kinase signaling pathways [4]. In clinical trials, linifanib has demonstrated anti-tumor activity in advanced solid tumors including non-small cell lung cancer (NSCLC), renal cell cancer, hepatocellular cancer, colorectal cancer, and breast cancer [5–11]. In a double-blind, randomized phase 2 trial, the addition of linifanib to carboplatin and paclitaxel resulted in significant improvement in response rates and progression-free survival in patients with advanced NSCLC [9].

A number of drugs have been developed to target specific tyrosine kinases known to be active in certain cancers. Some tyrosine kinases are essential for cardiac function, however. As a result, a side effect of TKI treatment has been development of cardiac events, such as a delay in cardiac repolarization [2, 12, 13]. Prolongation of the QT interval (duration of ventricular depolarization and subsequent repolarization) may increase the risk of torsade de pointes or other ventricular tachyarrhythmias. Although a number of TKIs have been associated with QT prolongation, the majority of TKIs do not lead to appreciable QT prolongation at clinical doses [14–17]. Many of these studies, however, have not been conducted in oncology patients, who may be at a greater risk due to concurrent or previous therapies. It was therefore the objective of this high-precision QT study to investigate the effect of linifanib on QT prolongation in patients with advanced solid tumors.

## Methods

A phase 1, single dose, open-label, randomized study in subjects with advanced solid tumors was conducted on 24 patients. This study was performed in accordance with the ethical standards laid down in the Declaration of Helsinki. All patients gave their informed consent prior to participation in the study. Eligibility included age >18 years, ECOG performance status scores of 0–1, and adequate organ function. For the assessment of ECGs, patients were randomly assigned to 2 sequences of regimens of linifanib at the maximum tolerated dose, 0.25 mg/kg, without exceeding 17.5 mg, administered orally in a two-period (Day 1 and Day 7) crossover fashion. Subjects were administered a single morning dose under fasting or non-fasting conditions.

A single 12-lead resting ECG was obtained within the week before Day 1, or on Day 1, and at study completion, or upon subject discontinuation. Triplicate ECGs were obtained serially on Day-1 at the anticipated time points for subsequent dosing and before and after dosing on Day 1 and Day 7 (crossover period 1 and period 2, respectively). The time points for measurements were pre-dose and 0.5, 1, 2, 3, 4, 6, 8, 10, 12, and 24 h post-dose. Measurements were taken after the subject had been supine for 5 min. Pharmacokinetic plasma samples were also collected for 72 h on Day 1 and Day 7.

QT, RR, PR, and QRS intervals were measured for each ECG using AbbVie’s validated PC-based algorithm (ABBIOS), with standardized manual over-reading of all ECGs by trained technicians and T–U morphology assessment by cardiologists. QTc was determined using Fridericia’s correction method (QTcF):

$${\text{QT}}_{F} = \frac{\text{QT}}{{\sqrt[3]{\text{RR}}}}$$

Values for the triplicate ECGs were averaged to obtain a single-interval measurement for each time point.

A linear mixed-effects model was used for the analysis of the Day 1 and Day 7 data to evaluate the effect of linifanib on cardiac repolarization. The analysis was performed for time-matched baseline-adjusted QTcF intervals (QTcF). For assessment of the effect of linifanib, the primary endpoint was the largest time-matched difference for QTcF between drug regimens and baseline (ΔQTcF). An intersection–union test was performed at a significance level of 0.05 within the framework of the corresponding mixed-effects model. Linifanib was considered to have a negative effect on cardiac repolarization if at all time points of the ECG measurements, the mean QTcF for linifanib, did not exceed the baseline mean by 10 ms or more with statistical significance level of 0.05. Therefore, the maximum 95 % upper confidence bound for the baseline-adjusted QTcF (ΔQTcF) must be less than 10 ms in order to demonstrate a negative QTc effect. The intersection–union test required high operational and statistical precision of the data to meet the criteria for negative QT effect, since the confidence intervals would be narrower with tighter variability.

Additionally, the relationship between baseline-adjusted QTcF and plasma drug concentration was explored using an exposure–response analysis. The equation for the response variable QTcF (Y) is:

$$Y = \, \mu \, + \alpha\,^{*} \,{\text{BASEQTcF}} + {\text{SEQUENCE}} + {\text{HOUR}} + {\text{DAY}} + \, \beta\,^{*}\, {\text{Concentration}} + \, \eta_{i} + \varepsilon_{ijk}$$

The model has terms for the baseline measurement (BASEQTcF), sequence (SEQUENCE), day of measurement (DAY), and time of measurement (HOUR). The random components of this model are denoted by *η*_*i*_ and *ε*_*ijk*_, with *i* identifying the *i*th subject, *j* identifying the day, and *k* identifying the time of the measurement within a day. Within the frame work of this model, the 95 % upper confidence bound for the effect of the mean Cmax of the linifanib dose on the QTcF was provided. If the bound is less than 10 ms, the regimen does not have a clinically relevant effect on cardiac repolarization.

## Results

Twenty-four subjects were included in the QTcF analysis. No subject had QTcF values greater than 500 ms, and no subject had a change greater than 60 ms from baseline. One subject had an asymptomatic QTcF change of greater than 30 ms from baseline.

### Intersection–union test

Among the study population, baseline QTcF values ranged from 360.9 to 468.6 ms. After patients received linifanib, the ΔQTcF for the fasting regimen ranged from −4.14 to 0.64 ms, whereas the non-fasting regimen ranged from −6.03 to −1.57 ms (Table 1). The maximum 95 % upper confidence bound for the drug effects for linifanib was 4.30 ms. These results are below the threshold of regulatory concern as indicated in ICH E14 Guidance for Industry [18]. It was therefore concluded that linifanib had no effect on cardiac repolarization.

**Table 1:** Intersection–union test results for linifanib on QTcF

Regimens	Time point (h)	QTcF Mean		Point^a^ Estimate	95 % Upper confidence bound
		Drug	Baseline		
Linifanib fasting regimen	0.5	421.8	423.9	−2.23	1.43
	1	422.0	423.1	−1.24	2.43
	2	422.1	421.4	0.57	4.24
	3	422.6	421.9	0.64	4.30
	4	418.1	422.4	−1.92	1.78
	6	415.8	419.9	−4.14	−0.47
	8	417.5	420.2	−2.82	0.85
	10	419.6	420.4	−0.58	3.12
	12	420.1	420.2	−0.16	3.50
	24	422.1	423.5	−1.53	2.14
Linifanib non-fasting regimen	0.5	419.8	423.9	−4.15	−0.48
	1	418.3	423.1	−4.89	−1.23
	2	417.1	421.4	−4.33	−0.67
	3	416.0	421.9	−6.03	−2.36
	4	413.8	422.4	−3.82	−0.12
	6	416.9	419.9	−3.05	0.61
	8	416.4	420.2	−3.87	−0.20
	10	417.2	419.4	−1.83	1.90
	12	417.3	418.9	−1.78	1.92
	24	421.0	422.4	−1.57	2.14

^a^ QTcF interval change form baseline (ΔQTcF) of the least squares means (msec)

### Exposure–response analysis

**Fig. 1:**
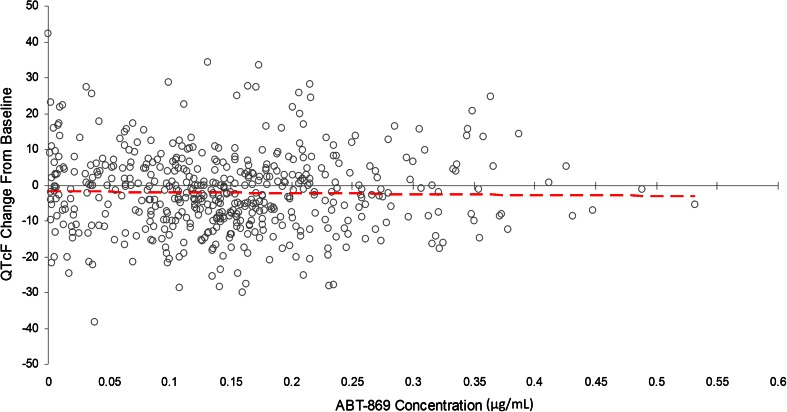
Linifanib concentration versus QTcF change from baseline (ΔQTcF)

Analysis was also performed with linifanib concentration as the drug exposure variable. The mixed-effects model showed a linear relationship between changes in QTcF interval and linifanib concentration (Fig. 1). The model estimated a slope of 0.01048 with a standard error of 0.006537 (*P* = 0.1094). This predicted a trend toward a change in QTcF interval of 3.56 ms at a concentration of 0.34 μg/mL (the Cmax at the maximum tolerated dose) and a 95 % upper confidence bound of 7.2 ms. In addition to supporting the finding that linifanib does not significantly affect QT interval, this model may provide useful predictions about the impact of other dosing regimens on QT prolongation.

### T–U waves morphological change

A morphological evaluation was performed for T and U waves at each ECG data collection time point. No clinically significant morphological changes in ECG, including no abnormal U waves, were observed following linifanib treatment. Isolated non-specific T wave abnormalities were seen and expected in patients who may have previously undergone cytotoxic cancer treatments.

## Conclusions

Maintaining cardiac function in patients undergoing cancer treatments is a concern in the development of any new drug. Advancements in molecular medicine have provided a number of attractive targets in the tyrosine kinase family of growth and proliferation signaling enzymes. In some cases, however, these drugs can interfere with cardiac repolarization and may pose a risk to patients who have undergone or are currently on cancer therapy. The current study is one of a few to rigorously test the effect of an investigational drug on cardiac repolarization in patients with advanced tumors who are refractory to standard treatments.

Analysis of the resulting data has concluded that linifanib does not pose a heightened risk for QTc prolongation in this refractory patient population. Despite a sample size of 24 subjects, the data had high operational and statistical precision as the 95 % upper confidence bounds for mean differences from baseline were below the threshold of regulatory concern at all time points. Exposure–response modeling showed QTcF change was not significant at the maximum concentration for the maximum tolerated dose, which further supports a lack of QT prolongation with linifanib. There were also no significant T or U wave morphological changes as determined by trained investigators. A categorical analysis of subjects with an absolute QTcF value in excess of 500 ms or change in baseline for more than 30–60 ms supports the absence of clinically significant effects. More broadly, at the time of this analysis, no significant adverse events related to abnormal cardiac repolarization were reported in this trial nor the concurrent phase 1, 2, and 3 clinical trials, representing an analysis of more than 700 linifanib-treated patients (data on file).

## References

[1] Krause DS, Van Etten RA (2005). Tyrosine kinases as targets for cancer therapy. N Engl J Med.

[2] Chen MH, Kerkela R, Force T (2008). Mechanisms of cardiac dysfunction associated with tyrosine kinase inhibitor cancer therapeutics. Circulation.

[3] Dai Y (2007). Discovery of N-(4-(3-amino-1H-indazol-4-yl)phenyl)-N’-(2-fluoro-5-methylphenyl)urea (ABT-869), a 3-aminoindazole-based orally active multitargeted receptor tyrosine kinase inhibitor. J Med Chem.

[4] Kieran MW, Kalluri R, Cho YJ (2012). The VEGF pathway in cancer and disease: responses, resistance, and the path forward. Cold Spring Harb Perspect Med.

[5] Wong CI (2009). Phase I and biomarker study of ABT-869, a multiple receptor tyrosine kinase inhibitor, in patients with refractory solid malignancies. J Clin Oncol.

[6] Tan EH (2011). Phase 2 trial of Linifanib (ABT-869) in patients with advanced non-small cell lung cancer. J Thorac Oncol.

[7] Tannir NM (2011). Phase 2 trial of linifanib (ABT-869) in patients with advanced renal cell cancer after sunitinib failure. Eur J Cancer.

[8] Toh HC (2013). Phase 2 trial of linifanib (ABT-869) in patients with unresectable or metastatic hepatocellular carcinoma. Cancer.

[9] Ramalingam SS et al (2012) Randomized phase II study of carboplatin and paclitaxel with either linifanib or placebo for advanced nonsquamous NSCLC. J Clin Oncol 30(15 Suppl):7512 10.1200/JCO.2014.55.7173 PMC5478045 25559798

[10] O’Neil BH et al (2012) Randomized phase II open-label study of mFOLFOX6 in combination with linifanib or bevacizumab for metastatic colorectal cancer. J Clin Oncol 30(15 Suppl):3532 10.1016/j.clcc.2014.04.001 25066269

[11] Cainap C et al (2013) Phase III trial of linifanib versus sorafenib in patients with advanced hepatocellular carcinoma (HCC). J Clin Oncol 31(4 Suppl):249 10.1200/JCO.2013.54.3298 PMC4279237 25488963

[12] Chu TF (2007). Cardiotoxicity associated with tyrosine kinase inhibitor sunitinib. Lancet.

[13] Kantarjian H (2006). Nilotinib in imatinib-resistant CML and Philadelphia chromosome-positive ALL. N Engl J Med.

[14] Abbas R (2012). A randomized, crossover, placebo- and moxifloxacin-controlled study to evaluate the effects of bosutinib (SKI-606), a dual Src/Abl tyrosine kinase inhibitor, on cardiac repolarization in healthy adult subjects. Int J Cancer.

[15] Tolcher AW (2011). A phase I open-label study evaluating the cardiovascular safety of sorafenib in patients with advanced cancer. Cancer Chemother Pharmacol.

[16] Sonnichsen D (2013). Analysis of the potential effect of ponatinib on the QTc interval in patients with refractory hematological malignancies. Cancer Chemother Pharmacol.

[17] del Corral A (2012). Midostaurin does not prolong cardiac repolarization defined in a thorough electrocardiogram trial in healthy volunteers. Cancer Chemother Pharmacol.

[18] Food and Drug Administration (2005) Guidance for industry: E14 clinical evaluation of QT/QTc interval prolongation and proarrhythmic potential for non-antiarrhythmic drugs 16237860

